# Supplementary material for: Pectin–Zeolite-Based Wound Dressings with Controlled Albumin Release
Source: Polymers (Basel). 2022 Jan 24;14(3):460. doi: 10.3390/polym14030460 (PMC8839484; doi:10.3390/polym14030460)
Supplement: Supplementary file 1 [file polymers-14-00460-s001.zip › polymers-1530716-supplementary.pdf]

Supplementary Material

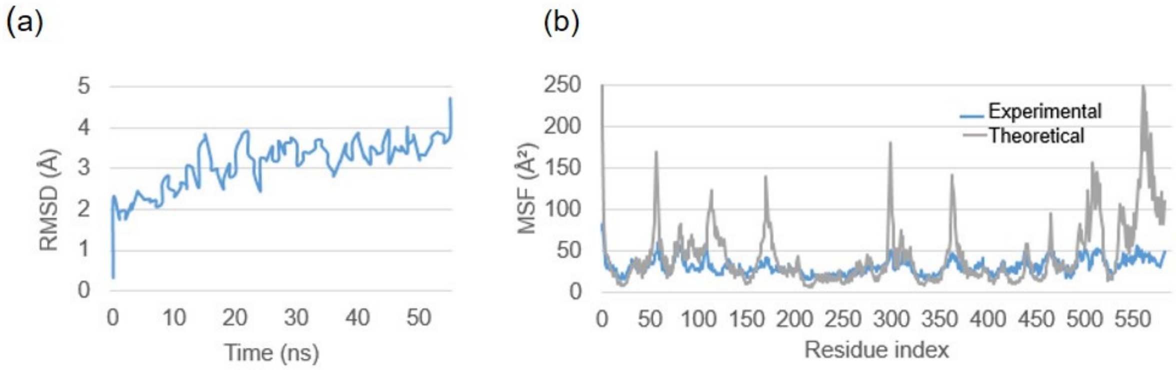

Figure S1. (**a**) Root mean squared deviation and (**b**) Mean squared fluctuations of human serum albumin with molecular dynamics simulations.
